# The Novel Phosphatase Domain Mutations Q171R and Y65S Switch PTEN from Tumor Suppressor to Oncogene

**DOI:** 10.3390/cells10123423

**Published:** 2021-12-05

**Authors:** Jose Antonio Ma. G. Garrido, Krizelle Mae M. Alcantara, Joshua Miguel C. Danac, Fidel Emmanuel C. Serrano, Eva Maria Cutiongco-de la Paz, Reynaldo L. Garcia

**Affiliations:** 1 Disease Molecular Biology and Epigenetics Laboratory, National Institute of Molecular Biology and Biotechnology, University of the Philippines Diliman, Quezon City 1101, Philippines; jose_antonio_ma.garrido@upd.edu.ph (J.A.M.G.G.); KrizelleMae.Alcantara@nationwidechildrens.org (K.M.M.A.); jcdanac@up.edu.ph (J.M.C.D.); fidel.serrano@bzh.uni-heidelberg.de (F.E.C.S.); 2 Institute of Human Genetics, University of the Philippines Manila, Manila 1000, Philippines; eccutiongcodelapaz@up.edu.ph; 3 Philippine Genome Center, University of the Philippines System, Diliman, Quezon City 1101, Philippines

**Keywords:** PTEN, colorectal cancer, EGFR pathway, tumor suppressor

## Abstract

Phosphatase and tensin homolog deleted on chromosome 10, or PTEN, is a well-characterized tumor suppressor with both lipid and protein phosphatase activities. PTEN is often downregulated by epigenetic mechanisms such as hypermethylation, which leads to constitutive activation of the PI3K–Akt pathway. Large datasets from next-generation sequencing, however, revealed that mutations in PTEN may not only hamper protein function but may also affect interactions with downstream effectors, leading to variable oncogenic readouts. Here, two novel PTEN mutations, Q171R and Y65S, identified in Filipino colorectal cancer patients, were phenotypically characterized in NIH3T3 and HCT116 cells, alongside the C124S canonical mutant and wild-type controls. The novel mutants increased cellular proliferation, resistance to apoptosis and migratory capacity. They induced gross morphological changes including cytoplasmic shrinkage, increased cellular protrusions and extensive cytoskeletal reorganization. The mutants also induced a modest increase in Akt phosphorylation. Further mechanistic studies will help determine the differential oncogenic potencies of these mutants, and resolve whether the structural constraints imposed by the mutations may have altered associations with downstream effectors.

## 1. Introduction

Phosphatase and tensin homolog deleted on chromosome 10, or PTEN, is a protein that is often mutated in a wide variety of cancers, including colorectal cancer (CRC). It functions as a regulator of the PI3K–Akt signaling pathway by dephosphorylating phosphatidylinositol (PI) 3,4,5-triphosphate to PI-4,5-bisphosphate, thereby interrupting the signaling cascade and halting function. Unperturbed, the PI3K–Akt signal transduction pathway promotes metabolism, proliferation, cell survival, growth, and angiogenesis in response to extracellular signals mediated by growth factors and cytokines such as epidermal growth factor, insulin, or interleukin-8, in various cellular contexts. With loss of PTEN function due to mutations, these processes may go unchecked and lead to a cancerous phenotype [1]. In the context of CRC, PTEN function has been found to be altered through both genetic and epigenetic mechanisms. Previous studies have demonstrated how deletion, point mutations, hypermethylation and altered subcellular location of PTEN are all closely correlated with carcinogenesis, disease progression, and prognosis of malignancy [2]. More recently, PTEN was shown to be subject to regulation by microRNAs, pseudogene molecular decoys, its antisense transcript *PTEN-AS*, and competitive endogenous RNAs [3,4,5,6,7].

PTEN is the second most commonly mutated tumor suppressor in solid tumors next only to p53 [8]. In CRC, low PTEN expression has been associated with several negative outcomes such as a more advanced tumor-node-metastasis (TNM) stage, higher incidence of lymph node metastasis, and an increased chance of local recurrence [9]. Aside from its role in oncogenesis, germline PTEN mutations have also been described in patients with the well-known PTEN hamartoma tumor syndromes, as well as several neurodevelopmental disorders, such as autism spectrum disorders and developmental delay [10]. While PTEN’s role in oncogenesis is most commonly attributed to its function as the primary negative regulator of the PI3K–mTOR signaling pathway, it has other functions in cell cycle regulation, migration and tumor progression that may play a part in the development of cancer.

As PTEN is considered a haploinsufficient tumor suppressor, partial loss of PTEN function is enough to promote tumor development, and even just a 50% reduction in PTEN levels is associated with the acceleration of cancer progression [11]. Studies using hypomorphic mouse models expressing reduced PTEN levels have shown that even just subtle aberrations in PTEN expression or functionality can significantly increase susceptibility to cancer. The loss of just one normal PTEN gene copy has been shown to cause a modest increase in Akt phosphorylation relative to normal cells, while an even greater increase in phosphorylation has been observed both in tissues and primary cells collected from heterozygous knock-in mice carrying just one normal and one mutant PTEN gene copy [12].

Previous studies have shown that PTEN mutations act in a dominant negative manner to suppress PTEN protein function [13]. PTEN adopts an active conformation as a homodimer and the catalytically inactive PTEN C124S as well as the lipid phosphatase-deficient mutant G129E may form heterodimers with wild-type PTEN to effectively constrain its phosphatase activity. This suggests that PTEN loss and PTEN mutations may not be synonymous in the context of cancer. Furthermore, this implies that patients harboring missense mutations in PTEN may be more susceptible to malignant cancer progression, arguing for PTEN mutational status as a potential prognostic marker for stratifying patients who may benefit from more radical interventions.

In this study, we report the phenotypic characterization of the novel PTEN phosphatase domain mutants Q171R and Y65S identified in a retrospective study of Filipino colorectal cancer patients at the University of the Philippines National Institutes of Health [14]. PTEN Q171R is curated in the Catalog of Somatic Mutations in Cancer (COSMIC; Genomic Mutation ID COSV64288519) database [15,16] and has been identified in two cases of breast cancer [17,18], one glioblastoma [19], one endometrial cancer [20], and one case of esophageal cancer [18], but has not been reported in colorectal cancer. It was classified as deleterious and pathogenic with a FATHMM (Functional Analysis through Hidden Markov Models) prediction score [21] of 0.96. Y65S has not been reported in the literature or in any publicly available database. Both mutants have not been previously characterized.

## 2. Materials and Methods

### 2.1. Generation of Wild-Type and Mutant PTEN Constructs

The full-length PTEN transcript variant 1 coding sequence (NM_000314.6) was reamplified using a pCMV-Flag-WT-PTEN construct [22] (gift from Hong Wu; Addgene plasmid #22231; http://n2t.net/addgene:22231; RRID Addgene_22231; Addgene, Watertown, Massachusetts, USA) as a template. The resulting amplicon was TA-cloned into the pTargeT™ mammalian expression vector (Promega Corporation, Madison, WI, USA). Primers for site-directed mutagenesis by overlap extension PCR were then designed to generate the C124S, Y65S, and Q171R mutant constructs. Primers used for PCR amplification are shown in Table 1.

The PCR reactions consisted of the following: 50 ng of template DNA, 1X Titanium^®^ Taq PCR buffer (Clontech Laboratories, Inc., Mountainview, CA, USA), deoxynucleoside triphosphates (Promega) at 0.125 μM each, forward (F) and reverse (R) primers at 2 μM each, and 1U Titanium^®^ Taq polymerase (Clontech Laboratories, Inc.). The PCR program used consisted of 94 °C initial denaturation for 5 min; 25–30 cycles of denaturation at 94 °C for 30 s, annealing at 55 °C for 30 s and extension at 72 °C for 30 s: and a final extension step at 72 °C for 10 min.

For site-directed mutagenesis, the overlapping 5′ and 3′ halves were generated by using inner mutagenic reverse and forward primers paired with the outer wild-type forward and reverse primers, respectively. The amplified halves from the first round of PCR were then mixed (25 ng of each fragment) and used as a template for splicing by overlap extension PCR to generate the full-length PTEN mutant construct, using the wild-type PTEN outer forward and reverse primers and PCR conditions as stated above. Mutant amplicons were directly TA-cloned into the pTargeT™ vector. All constructs were verified error-free by Sanger sequencing.

### 2.2. Cell Culture and Transfection of NIH3T3 and HCT116 Cells

Choice of the murine embryonic fibroblast cell line NIH3T3 (Cat. No. CRL-1658; American Type Culture Collection (ATCC), Manassas, VA, USA) for cell proliferation, wound healing, cytoskeletal staining, and gross morphology assays was guided by the following considerations: (a) The cell line has routinely been used for characterizing wild-type and mutant oncogenes and tumor suppressors, because of its advantage of not requiring complementary cooperative mutations for oncogenic transformation to manifest [23,24]. (b) Unlike NIH3T3 which has a wild-type background, commonly used CRC cell lines have background mutations in RAS or PIK3CA which can mask the phenotypic readouts of the oncogene or tumor suppressor under study, e.g., HCT116 (KRAS G13D and PIK3CA H1047R), DLD-1 (KRAS G13D), SW480 (KRAS G12V), and SW948 (KRAS Q61L and PIK3CA E542K). (c) Lastly, and most importantly, PTEN expression is known to be downregulated by oncogenic Ras [25]. Hence, effects of the PTEN mutants under study may be dampened or cannot easily be decoupled from the effects of a mutant Ras background for particular cancer hallmark assays. For apoptosis assays, HCT116 (Cat. No. CCL-247, ATCC) was used since the inducer sodium butyrate triggers senescence instead of apoptosis in NIH3T3 cells [26]. Although resistance to apoptosis can be measured in this cell line with a mutant Ras and PIK3CA background, differences in promoting cell survival among wild-type and mutant setups may not easily be resolved [27].

As per the manufacturer’s recommendations, Dulbecco’s modified Eagle’s medium (DMEM; Gibco, Thermo Fisher Scientific, Inc., Waltham, MA, USA) supplemented with 10% newborn calf serum (NBCS; Gibco, Thermo Fisher Scientific, Inc.), with 100 U/mL penicillin/streptomycin and 3.7 g/L sodium bicarbonate added, was used to culture NIH3T3 cells. Roswell Park Memorial Institute 1640 medium (RPMI-1640; Gibco, Thermo Fisher Scientific, Inc.) supplemented with 10% fetal bovine serum (FBS; Gibco, Thermo Fisher Scientific, Inc.), and with 50 U/mL penicillin/streptomycin and 2.0 g/L sodium bicarbonate added, was used to culture HCT116 cells. All cells were cultured in 75 cm^2^ cell culture flasks and maintained in a humidified incubator at 37 °C with 5% CO_2_. The cells were observed daily and were split when confluence reached around 90%.

NIH3T3 and HCT116 cells were seeded at their corresponding cell concentrations and well/plate formats as indicated in the individual assays. For each assay, cells were transfected with an appropriate amount of the mutant constructs or pTargeT^TM^ empty and PTEN wild-type controls. FuGENE^®^ HD transfection reagent (Promega) and Lipofectamine^®^ 2000 (Invitrogen; Thermo Fisher Scientific, Inc., Carlsbad, CA, USA) were used for NIH3T3 and HCT116 cells, respectively. To monitor transfection efficiency, control setups were transfected with an equivalent amount of the pmR-ZsGreen1 reporter vector which expresses the green fluorescent protein ZsGreen1 (pmR-ZsGreen1, Clontech Laboratories, Inc.), and visualized by fluorescence microscopy. Transfection protocols were optimized to achieve at least 70% efficiency between 24 and 72 h post-transfection for all assays.

### 2.3. Western Blot Analysis

NIH3T3 cells were seeded at 150,000 cells/well in 12-well plates and transfected with 1000 ng plasmid DNA after 24 h. At 24 h post-transfection, cells were switched to DMEM +0.5% NBCS. At 48 h post-transfection, cells were treated with 2 ng/mL epithelial growth factor (EGF) for 5 min, before cell lysis by incubating in 30 µL RIPA lysis buffer supplemented with protease inhibitors (RIPA+) for 5 min on ice with occasional swirling. Lysate was briefly vortexed and clarified by centrifugation at 10,000× *g* for 20 min. Total protein was quantified through the bicinchoninic acid (BCA) assay. Around 20 µg of total protein was diluted with 10 µL RIPA+ buffer and denatured in SDS-PAGE sample buffer by boiling at 99°C for 5 min. Samples were electrophoresed on an Any kD™ Mini-PROTEAN^®^ TGX Stain-Free™ Precast Gel (Bio-Rad) at 120 V for about 1 h. Transfer to a PVDF membrane was then carried out using the Trans-Blot^®^ Turbo™ Transfer System (Bio-Rad) at 1.3 A, up to 25 V, 7 min. Blots were blocked in 5% BSA in 1X Tris-buffered saline with 0.2% Tween-20 (TBST). Primary antibody (1:1000 rabbit anti-PTEN pAb, Invitrogen 51-2400; 1:1000 rabbit anti-phospho-Akt (Ser473), Cell Signaling Technology #4060; or 1:1000 mouse anti-(pan) Akt, Cell Signaling Technology #2920) was incubated overnight at 4°C, followed by three 5 min washes in TBST, and 1 h incubation at room temperature with secondary antibody (1:10,000 goat anti-rabbit IgG, HRP-conjugated, Invitrogen 31460). Blot was then washed thrice in TBST for 5 min and twice in TBS for 5 min. Blot was then incubated in Luminata Classico Western HRP Substrate (Merck) for 2 min and then imaged with the ChemiDoc™ Imaging System (Bio-Rad). To probe successive targets, the blot was stripped by incubation in mild stripping buffer (1.5% glycine, 0.5% SDS, 1% Tween-20, pH 2.2) for 15 min, washed thrice with TBST for 5 min, and then re-blocked with 5% BSA in TBST overnight. For the loading control, after stripping, probing was then carried out by incubation in primary antibody (1:4000 rabbit anti-GAPDH mAb, Cell Signaling Technology 2118S) for 1 h at room temperature, and the subsequent steps of the protocol as above. Densitometric quantification was performed using Image Lab v6.0.1 software (Bio-Rad).

### 2.4. MTS-Based Cell Proliferation Assay

In 96-well plates, NIH3T3 cells were seeded at 2500 cells/well. At 24 h post-seeding, cells were transfected with 200 ng of each pTargeT^TM^ construct in triplicate, after which they were maintained in DMEM media supplemented with 10% NBCS. At 48 and 72 h post-transfection, 10 µL of CellTiter 96^®^ Aqueous One-Cell Proliferation Assay Reagent (Promega) was added to each well and incubated at 37 °C for 1 h. For each setup, absorbance values were measured at λ = 460 nm using a colorimetric plate reader (FLUOstar Omega Microplate Reader, BMG LABTECH, Cary, NC, USA). Using serial dilutions of an untransfected cell suspension at 2500, 5000, 10,000, 20,000, and 40,000 cells, a standard curve was generated by plotting the number of cells vs. A460. The mean cell counts were calculated per setup at each timepoint.

### 2.5. Caspase-3/7-Based Apoptosis Assay

In 96-well plates, HCT116 cells were seeded at 10,000 cells/well and transfected in triplicate, as described above. Two sets of three wells each per construct were prepared as either an induced or uninduced setup. The transfected cells were then incubated in RPMI media supplemented either with 4% FBS alone, or with 6 mM sodium butyrate for apoptosis induction. At 24 h post-induction, 10 µL of Caspase-Glo^®^ 3/7 Assay reagent (Promega) was added to each well. The plates were incubated for 2 h with gentle shaking at ambient temperature. Mean luminescence readings per setup were then measured with the FLUOstar Omega Microplate Reader.

### 2.6. Alexa Fluor^TM^ Annexin V Apoptosis Assay and Flow Cytometry

HCT116 cells were seeded at 50,000 cells/well in 12-well plates and transfected with 500 ng of each pTargeT^TM^ construct. After 24 h of incubation, the cells were harvested and resuspended in 1 mL of RPMI media. An aliquot of 100,000 cells was taken from each setup and transferred to a fresh 1.5 mL tube. The cells were spun at 2000× *g* and washed with ice-cold PBS thrice. The supernatant was removed and 100 µL of 1X binding buffer was added. Cells were stained by adding 2.5 µL Alexa Fluor 488 Annexin V (Thermo Fisher Scientific, Inc.) and 1.0 µL propidium iodide (PI). Annexin V binds to the exposed phosphatidylserine and by conjugating a fluorescent molecule to it, the quantification of apoptotic cells is possible with a flow cytometer. Propidium iodide (PI), a fluorescent intercalating agent that binds DNA, was added to rule out cells that were simply necrotic and not undergoing apoptosis [28]. After 15 min of incubation at room temperature, another 200 µL of 1X Binding Buffer was added. The stained cells were incubated on ice for 10 min and the cell counts were read using an Attune NxT flow cytometer (Thermo Fisher Scientific, Inc.). Control setups with unstained living cells and heat-killed cells with either stain were also prepared and read.

### 2.7. Wound-Healing Migration Assay

NIH3T3 cells were seeded in 96-well plates in triplicate and transfected as described above. The transfected cells were incubated for 48 h after transfection. Using sterile toothpicks, a thin artificial wound was scratched onto the center of transfected cell monolayers at >90% confluence. Cells were then maintained in DMEM supplemented with 10% NBCS since a tumor suppressor was being studied and dampening mitogenic stimulation may not reveal the tumor-suppressive effect of the reference wild-type construct. Wound closure was monitored for 20 h, each time capturing the same field of view per well per setup at 1 h intervals and at x10 magnification using an Olympus IX83 fluorescence microscope with a motorized stage and single plate incubator attachment (Olympus Corporation, Tokyo, Japan). Percent open wound area at 0, 6, and 12 h time points was determined using the TScratch software [29]. The rate (% wound area/time) was calculated as follows. The % wound area was defined as the area from each time point represented/area at t = 0 h (average of 3 field views per time point). The values were then plotted with the % wound area in the y-axis, and time in the x-axis. At t = 0, all of the setups will have 100% wound area, and subsequent timepoints have decreasing % wound area values. The line equation for each setup was calculated, with the slope representing the rate of wound closure. Images at 0, 6, 12, and 20 h time points were selected for visualization, but measurements were taken every hour.

### 2.8. Observation of Gross Morphology

In 24-well flat-bottom plates, NIH3T3 cells were seeded at 10,000 cells/well and transfected with 500 ng of each pTargeT^TM^ construct 24 h post-seeding. Transfected cells were monitored for changes in morphology (i.e., size, refringency or the presence of a ring of light around the cell periphery, presence of filopodia, presence of lamellipodia, and depolarization or rounding of the cell) using an inverted brightfield microscope (Olympus IX71, Olympus Corporation, Tokyo, Japan) at 40× magnification 72 h post-transfection. For each setup, the percentage of cells exhibiting transformed characteristics was determined in three fields of view. Those with altered morphology were counted for each documented field. Total cell count per view was then performed. Mean percentage of morphologically altered cells was computed for all three fields of view then statistically compared among all setups.

The Simple Neurite Tracer plugin of the Fiji software [30] was used to measure the length of cellular protrusions. Tracing was carried out from the center of the nucleus to the tip of the extension. The average length of protrusions for each setup was determined by dividing the total length of all protrusions with the total cell count, as determined via the cell counter plugin of ImageJ software [31]. The values were then compared among all setups.

### 2.9. Actin Cytoskeleton Staining

Using Millicell^®^ EZ 8-well chamber slides (Merck KGAa, Darmstadt, Germany), NIH3T3 cells were seeded at 6500 cells/well and transfected with 300 ng of each pTargeT^TM^ construct 24 h after seeding. After 48 h, transfected cells were fixed with 4% paraformaldehyde for 20 min on ice, then permeabilized with 0.1% Triton X-100 in 1X PBS for 15 min at room temperature, and washed thereafter with 1X PBS. The cells were then blocked with 1% BSA in PBS for 20 min at room temperature, and incubated in a 1:100 dilution of tetramethylrhodamine-conjugated phalloidin (Invitrogen; Thermo Fisher Scientific, Inc.) in 1X PBS for 1 h with gentle shaking at room temperature. After another wash with 1X PBS, nuclei were counterstained with Hoechst 33258 (1 µg/µL) for 5 min at room temperature. After the final washing step in 1X PBS, cells were mounted using SlowFade^TM^ Diamond antifade mountant (Invitrogen; Thermo Fisher Scientific, Inc.) and visualized under a fluorescence microscope (IX83, Olympus Corporation). A red fluorescent filter (λex/λem: 490/525 nm) was used to visualize filamentous actin structures, and a blue fluorescent filter (λex/λem: 355/465 nm) to visualize the nuclei.

### 2.10. Bioinformatics-Based Analysis of Potential Functional Impact of PTEN Mutations

The predicted functional effects of each novel PTEN mutation were analyzed using the bioinformatics programs Polymorphism Phenotyping (POLYPHEN-2; version 2, Harvard Genetics, Cambridge, MA, USA) [32], Sorting Intolerant from Tolerant (SIFT; version 5.2.2, Bioinformatics Institute, Singapore, Republic of Singapore) [33], and Align GVGD (Huntsman Cancer Institute, University of Utah, Salt Lake City, UT, USA) [34]. These programs were selected due to their ability to predict the effects of single amino acid substitutions using structural and phylogenetic data. The amino acid sequence for the wild-type PTEN protein was inputted into each program and the residues to be substituted were subsequently indicated.

The predicted effects of each non-canonical/novel PTEN mutation were also analyzed by observing how each individual mutation affects the overall structure of the PTEN protein. The protein structure of each PTEN mutant was first generated based on their DNA sequence using the wild-type PTEN (PDB ID: 5BUG) as a reference structure on the SWISS-MODEL web-based protein modelling platform (Swiss Institute of Bioinformatics, Basel, Switzerland) [35]. The mutant protein structures were then each overlaid onto the native PTEN structure using the BIOVIA Discovery Studio program (Datassault Systems, Inc., BIOVIA, San Diego, CA, USA) and root mean square deviation (RMSD) was calculated using the program.

### 2.11. Statistical Analysis

To measure differences between two setups, the unpaired two-tailed t-test was used for statistical analysis of the data. To test significant differences among multiple setups, analysis of variance with post hoc Tukey’s honest significant difference (HSD) was used. Data from quantitative experiments are presented as mean ± standard deviation. For all tests, significance was defined as * *p* < 0.05; ** *p* < 0.01; and *** *p* < 0.001.

## 3. Results

### 3.1. PTEN Mutants Q171R and Y65S Promote Proliferation in NIH3T3 Cells

**Figure 1:**
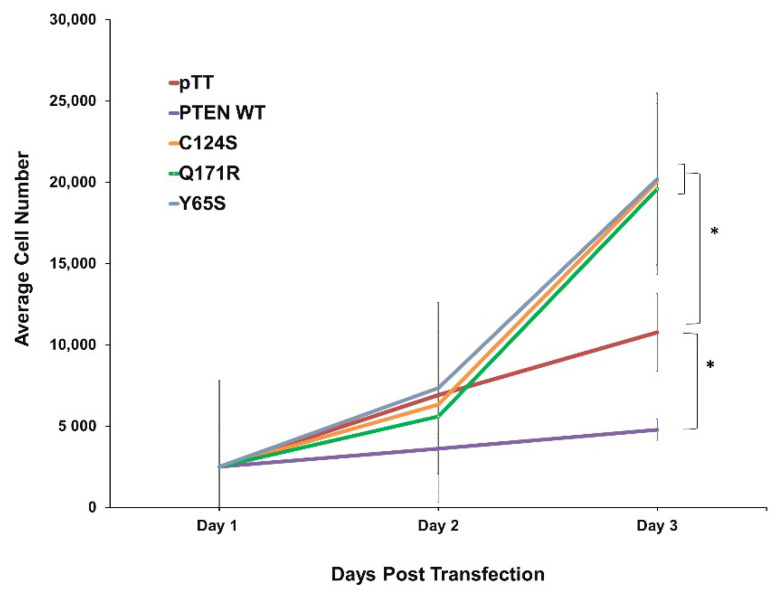
Expression of the novel PTEN mutants Q171R and Y65S enhance cell proliferation in NIH3T3 cells. Proliferation rate of NIH3T3 cells transfected with empty vector, wild-type PTEN, novel PTEN mutants, and canonical PTEN mutant. Data presented are representative of three independent trials in triplicate and expressed as mean ± standard deviation. * *p* < 0.05. WT = wild-type.

To determine the possible effects of the novel PTEN mutants Q171R and Y65S on cell proliferation, NIH3T3 cells transfected with each mutant construct were seeded in triplicate and in equal cell numbers onto 96-well plates. Over the course of 72 h, overexpression of either novel mutant significantly increased the average cell number, and thereby proliferation rate, of NIH3T3 compared to setups transfected with the wild-type PTEN or vector-only controls across three trials (Figure 1). The effect of both mutants is comparable to that of the canonical mutant, PTEN C124S, which likewise caused a significantly higher rate of proliferation.

### 3.2. PTEN Mutants Q171E and Y65S Confer Resistance to Apoptosis in HCT116 Cells

**Figure 2:**
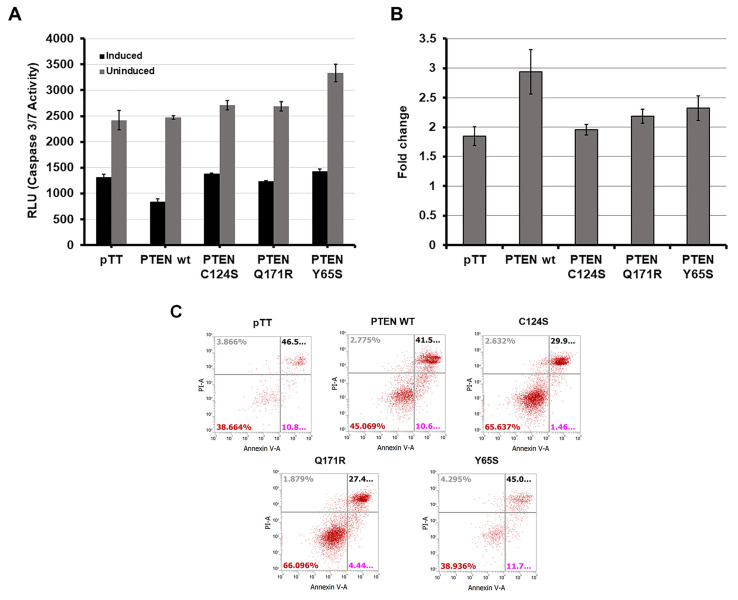
Expression of the novel PTEN mutants Q171R and Y65S enhance resistance to apoptosis in HCT116 cells. (**A**) Caspase-3/7 activity in sodium-butyrate-induced HCT116 cells transfected with empty vector, wild-type PTEN, or PTEN canonical and novel mutants. (**B**) Fold change in caspase activity between induced and uninduced setups showing ability of each construct to impede sodium-butyrate-induced apoptosis. Data presented are representative of three independent trials in triplicate and expressed as mean ± standard deviation. (**C**) Expression of PTEN mutant Q171R reduced the number of apoptotic HCT116 cells after induction with sodium butyrate. Apoptotic cells were identified through annexin V staining and counted using flow cytometry. WT = wild-type. RLU = Relative Luminescence Units.

Since the primary readouts of the PI3K–mTOR pathway are cell growth and survival, one of the more important cancer hallmarks to monitor in a mutant PTEN variant is its effect on apoptosis [36]. Two separate assays, the Caspase-Glo 3/7 assay and Annexin V staining (Figure 2), were conducted to assess how the novel PTEN mutants Q171R and Y65S would affect apoptosis. In both experiments, HCT116 cells overexpressing each variant were treated with the apoptosis inducer sodium butyrate (NaB) at 24 h post-transfection.

#### 3.2.1. Caspase-Glo 3/7 Assay

In the Caspase-Glo 3/7 assay, the observed luminescence corresponds proportionately to caspase-3/7 activity and, in turn, apoptotic events [37]. The luminescence readings from the uninduced and induced setups for each construct were compared to determine how overexpression of each could impede sodium butyrate’s ability to induce apoptosis. Overexpression of the novel PTEN mutants Q171R and Y65S in HCT116 cells consistently negated the increase in caspase-3/7 activity that would normally be observed as a consequence of overexpressing normal functioning PTEN, akin to the effect of the canonical PTEN mutant C124S (Figure 2A,B).

#### 3.2.2. Annexin V Staining Flow Cytometry

Annexin V staining was also employed to observe apoptotic activity. In this assay, overexpression of the PTEN variant Q171R consistently resulted in a lower number of HCT116 cells undergoing either early (Annexin V positive, propidium iodide negative) or late apoptosis (Annexin V positive, propidium iodide positive) compared to the empty vector or wild-type controls in three independent trials, thus implying resistance similar to the effect of the canonical mutant C124S. The PTEN variant Y65S did not show a significant effect on apoptotic activity in 2 out of 3 trials (Figure 2C). Overall, Q171R was observed to confer significant resistance to apoptosis in both apoptosis assays. Y65S, on the other hand, was observed to consistently result in resistance to apoptosis as observed through caspase-3/7 activity but not through Annexin V staining.

### 3.3. The Novel PTEN Mutants Q171E and Y65S Increase Migration Rate of NIH3T3 Cells

**Figure 3:**
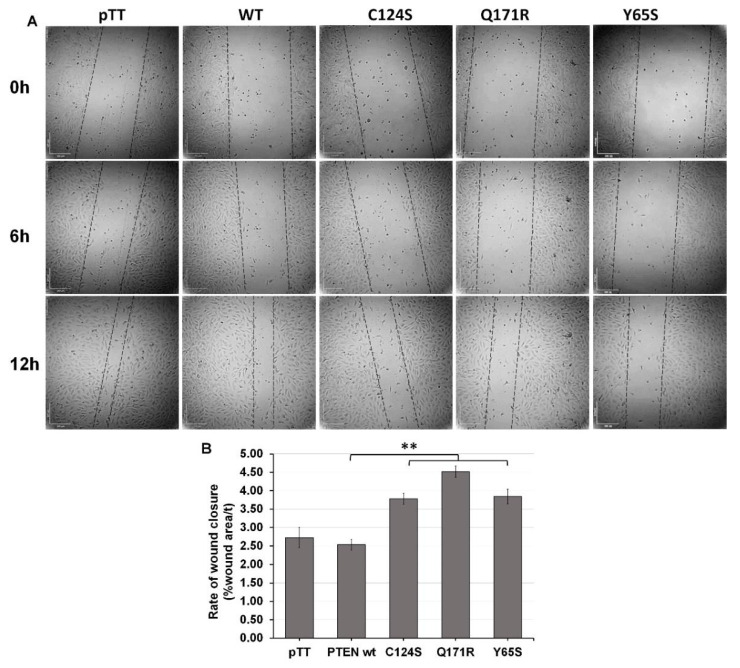
Expression of PTEN mutants enhance the migration rate of NIH3T3 cells. (**A**) Representative micrographs of NIH3T3 cells transfected with PTEN constructs at 0, 6 and 12 h after scratching the cell monolayers. (**B**) Quantification of wound area per hour showed that NIH3T3 cells overexpressing mutant PTEN covered more distance per hour than control cells. Data presented are representative of three independent trials in triplicate and expressed as mean ± standard deviation. ** *p* ≤ 0.01. Scale bars: 100 µm. WT = wild-type.

Wound healing assays were performed to investigate the effects of the novel PTEN mutants Q171R and Y65S on the migratory capacity of NIH3T3 cells. Photographs of each well were taken every hour for 20 h after an artificial wound was created with a sterile toothpick. The area of the wound that closed at the 0, 6, and 12 h marks was measured and used to compute the rate of wound closure. A computer-controlled motorized stage was used in order to ensure that the exact same area on each well was observed at every time point. Cells overexpressing the novel PTEN mutants each showed a significant increase in migration rate compared to wild-type PTEN and empty vector controls, akin to rates observed in cells expressing the canonical PTEN C124S variant (Figure 3).

### 3.4. The Novel PTEN Mutants Q171E and Y65S Promote Cell Rounding, Refringency, and Cytoplasmic Shrinkage

**Figure 4:**
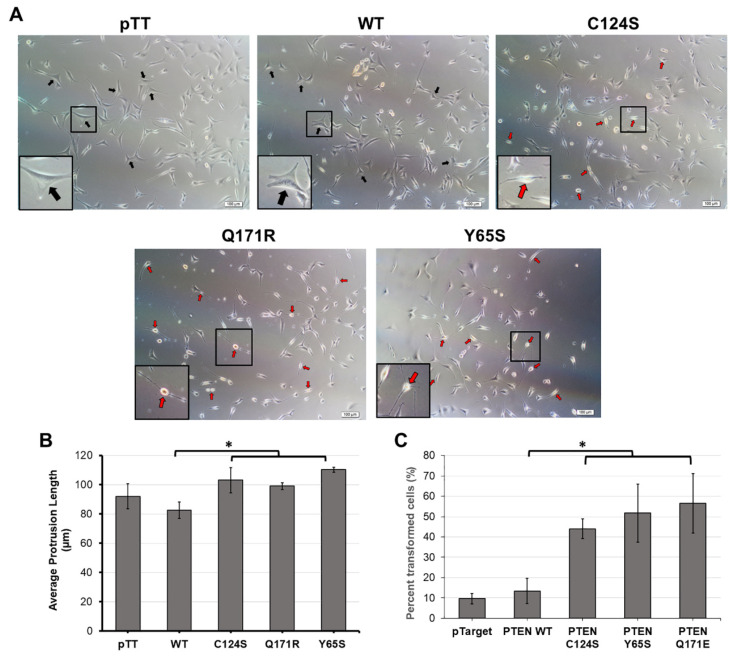
Overexpression of PTEN mutant constructs significantly alters gross cell morphology of NIH3T3 cells. NIH3T3 cells transfected with empty vector, wild-type, or mutant PTEN expression constructs were viewed at 20× magnification. Quantification of each trial was conducted in triplicate and representative pictures from each are shown above. (**A**) NIH3T3 cells displaying typical fibroblastic morphology (black arrows) are present in the empty vector setups as well as in cells transfected with wild-type PTEN constructs. In contrast, cells displaying the more oncogenic phenotype (red arrows) with round shrunken cytoplasm and clear protrusions were observed in NIH3T3 cells transfected with PTEN canonical and novel mutant constructs. Insets show zoomed in images of a representative cell from each setup. (**B**) Average length of cellular protrusions measured from the center of the nucleus to the tip of the extension. (**C**) Quantification of cells showed a marked increase in cells displaying transformed morphology in setups overexpressing the mutant PTEN constructs. Data presented are representative of three independent trials in triplicate and expressed as mean ± standard deviation. * *p* < 0.05. Scale bars: 50 µm. WT = wild-type.

Cellular morphology is greatly impacted during oncogenic transformation. Normal NIH3T3 cells are expected to exhibit a flat and well-spread morphology which is indicative of the established adhesion of cells to the culture substrate [38]. Transformed NIH3T3 cells, on the other hand, become smaller, round, birefringent, and with much longer protrusions [39,40,41]. To determine if the PTEN mutants Q171R and Y65S can also induce changes in the overall morphology of NIH3T3 cells, transfected cells overexpressing these mutant proteins were observed under a brightfield microscope. No discernible morphological changes were observed in cells transfected with the wild-type PTEN compared to empty vector control. On the other hand, cells expressing either PTEN Q171R or Y65S showed morphological changes and many were round, shrunken and more refringent, which is characteristic of transformed fibroblasts (Figure 4A). NIH3T3 cells with apparent morphological alterations such as decreased size, refringency, and increased cellular protrusions were then quantified (Figure 4B,C). Quantification confirmed that a significant fraction of fibroblasts transfected with the PTEN variants Q171R and Y65S, as well as the positive control C124S, exhibit these features characteristic of highly motile cells. These findings are supported by the results in the next section, showing extensive cytoskeletal reorganization revealed by actin staining of cells expressing the novel PTEN mutants, thereby reinforcing evidence of the transformative effect of the rare mutations on cellular morphology.

### 3.5. Cells Expressing the Novel PTEN Mutants Q171E and Y65S Display Altered F-Actin Cytoskeletal Organization

**Figure 5:**
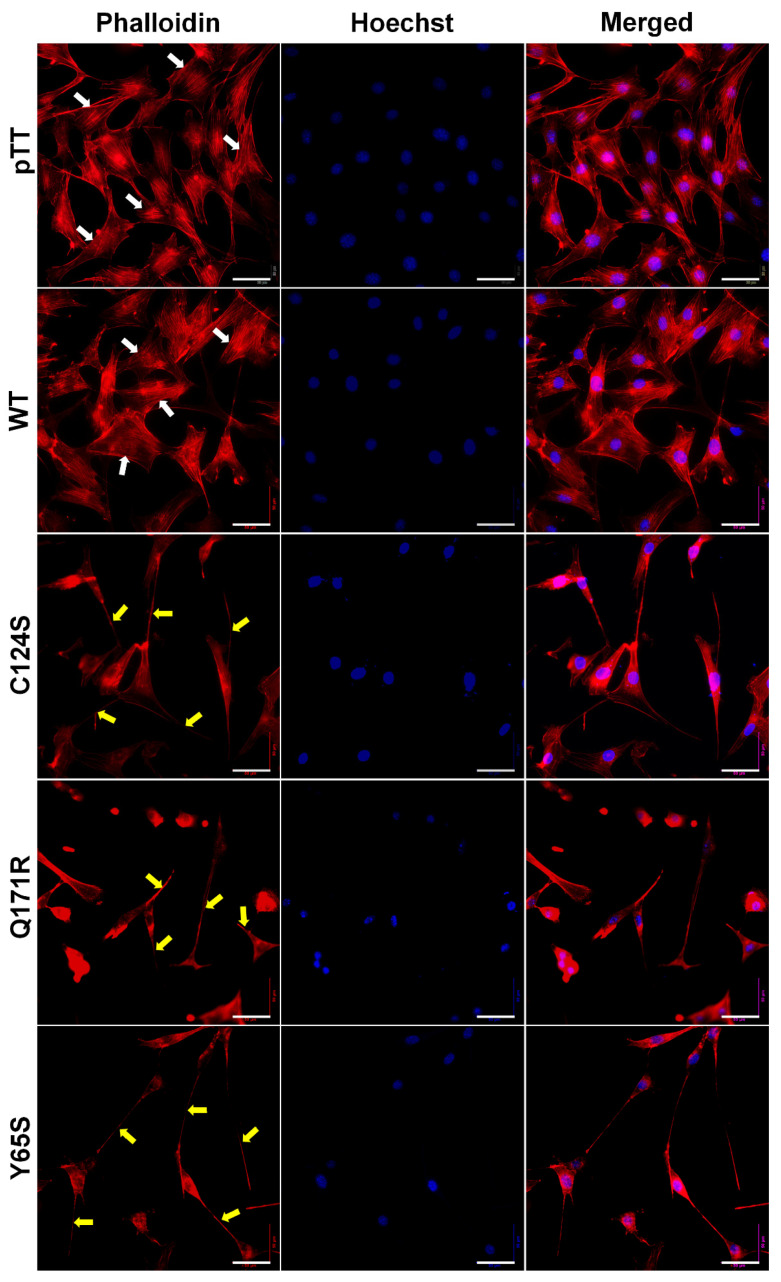
Expression of PTEN mutants alters the cytoskeletal organization of NIH3T3 cells. Fluorescent images taken at 40× magnification showing the F-actin cytoskeletal organization of NIH3T3 cells transfected with empty vector, WT PTEN, or PTEN mutant expression constructs. Prominent and highly organized stress fibers (white arrows) are present in the empty vector setups as well as in cells transfected with WT PTEN constructs. In contrast, long and slender cellular protrusions (yellow arrows) were more apparent in NIH3T3 cells transfected with PTEN canonical and novel mutant constructs. Cytoplasmic shrinkage and more diffused cytoskeletal actin were also observed in the mutant setups (TRITC-conjugated phalloidin: F-actin; Hoechst: nuclei). Scale bars: 25 µm. WT = wild-type.

Cytoskeletal reorganization is a typical indicator of more motile cells. Thus, the filamentous actin of fixed and permeabilized NIH3T3 cells overexpressing the novel PTEN mutants Q171R and Y65S was visualized through fluorescence-conjugated phalloidin staining (Figure 5).

Cells transfected with empty vector control and wild-type PTEN exhibited flat angular morphology with well-oriented parallel stress fibers typical of normal NIH3T3 cells. Cells overexpressing the PTEN Q171R and Y65S mutants, on the other hand, all displayed gross morphological changes in cytoskeletal organization, as evidenced by dramatic cytoplasmic shrinkage and changes in shape. Very long and slender cellular protrusions were also evident, with some extending from cell to cell and resembling tunneling nanotube-like structures. The morphological changes observed in cells expressing Q171R and Y65S were at least partially similar to those induced by the canonical mutant C124S, although cells with more obvious fan-shaped migrating front were more observable in the latter.

### 3.6. PTEN Mutants Increase Akt Phosphorylation in NIH3T3 Cells

**Figure 6:**
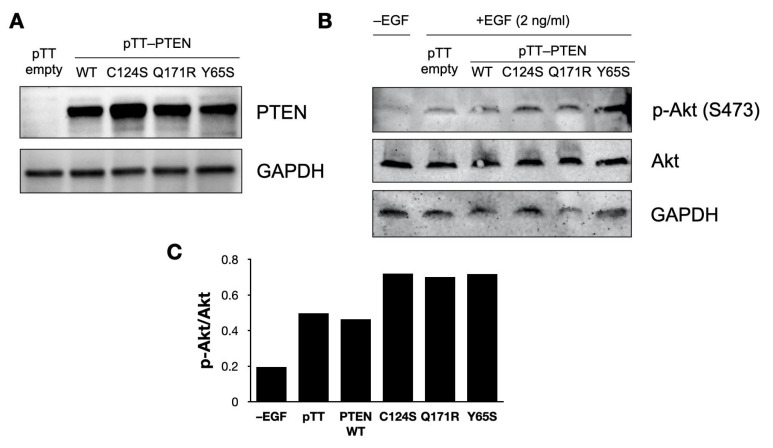
Western blotting to detect PTEN overexpression and Akt phosphorylation in NIH3T3 cells. (**A**) Confirmation of PTEN overexpression in transfected NIH3T3 cells; the empty vector control is shown. (**B**) Akt phosphorylation in NIH3T3 cells. Cells were serum-starved for 24 h before induction of Akt phosphorylation via a brief 5 min treatment with 2 ng/mL EGF, before harvesting protein lysates. (**C**) Densitometric quantification of Akt phosphorylation in (**B**), shown as the ratio of phosphorylated to total Akt. EGF = epithelial growth factor; pTT = pTarget vector; WT = wild-type.

As PTEN is a negative regulator of the PI3K–Akt pathway, mutants that impair the tumor suppressive function of PTEN might be expected to cause increased activation of this oncogenic signaling pathway. Accordingly, Akt phosphorylation in NIH3T3 cells was detected via Western blotting (Figure 6).

Successful overexpression of the PTEN constructs was confirmed (Figure 6A and Figure S1) and Akt phosphorylation was successfully induced by treating NIH3T3 cells with epithelial growth factor (EGF) (Figure 6B and Figure S1). Notably, cells overexpressing the PTEN mutants showed a modest increase in levels of Akt phosphorylation compared to the wild-type controls, and this was confirmed by densitometric analysis of the Western blots (Figure 6C).

### 3.7. Bioinformatics-Based Modeling of the PTEN Mutants Predict Their Oncogenic Impact

The possible effects of the PTEN mutants Q171R and Y65S on PTEN function were assessed using the online bioinformatics prediction tools SIFT, PolyPhen-2, and Align GVGD. SIFT analyzes sequence homology and the physical properties of amino acids to predict whether an amino acid substitution affects protein function [33]. PolyPhen-2 uses physical and comparative considerations to predict the impact of an amino acid substitution based on the protein’s structure and function [32]. Align GVGD classifies missense substitutions on a scale ranging from enriched deleterious to enriched neutral by combining the biophysical characteristics of amino acids and protein multiple sequence alignments [34]. Altogether, these scores are based on several features such as the primary amino acid sequence change, phylogenetic background, and possible structural change resulting from the mutation. The results of these simulations are presented in Table 2. As expected, the C124S canonical mutant garnered high SIFT, PolyPhen-2, and Align GVGD scores due to its position on the PTEN active site.

For the Q171R and Y65S mutations, the programs predicted that both would probably be damaging to PTEN protein function. The high predicted scores may be because both mutations lie within the annotated PTEN phosphatase domain (AA 7–185) and may thus interfere with its enzymatic activity. It is worth noting that mutations in residues 155–174 have been found to result in phosphatase-deficient mutants. Structural analysis of PTEN has revealed that residues 160–171 comprise a TI loop which contributes to substrate localization and fixation towards the active site [42], and thus may account for the predicted damaging contribution of the Q171R mutation on PTEN function. Additionally, residues that comprise PTEN’s catalytic loop include D92, C124, and R130 [42]. Due to its proximity to the D92 residue, the Y65S mutation may thus be capable of affecting PTEN catalytic function.

**Figure 7:**
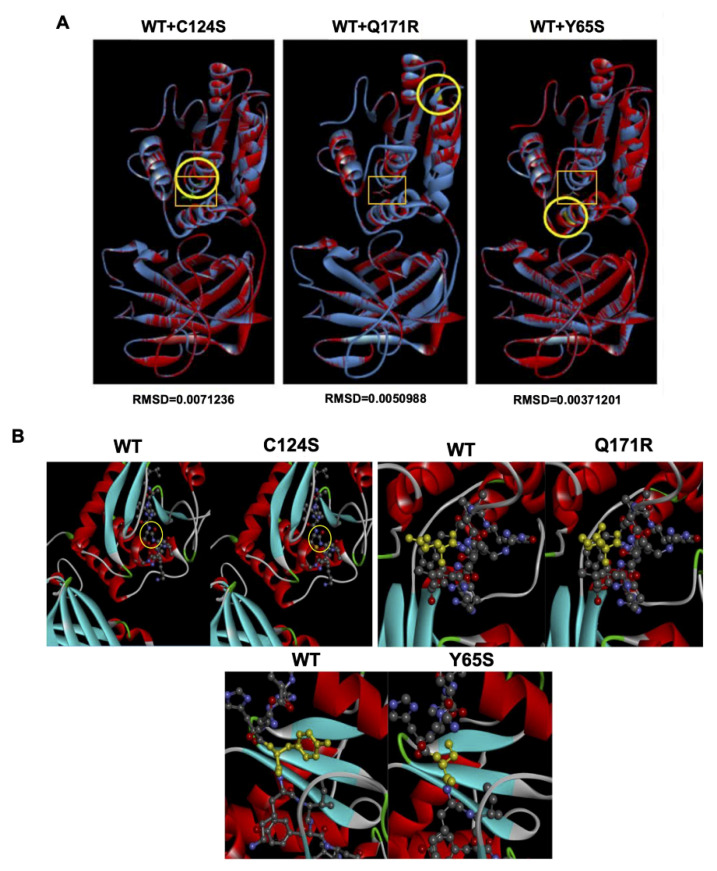
Bioinformatics-based modeling of the PTEN mutants predicts their oncogenic impact. (**A**) The canonical PTEN C124S mutant and novel PTEN Q171R and Y65S mutants (red) superimposed with the PTEN wild-type variant (blue) using the Biovia Discovery Studio bioinformatics prediction platform. The models were generated based on the PTEN (PDB ID: 5BUG) crystal structure as reference using the SWISS-MODEL web-based protein modeling platform. The position of each mutated amino acid is indicated by the yellow encircled residues and the active site of the protein is highlighted with an orange box. RMSD values of each PTEN mutant model versus PTEN WT as calculated using the BIOVIA Discovery Studio Visualizer are also indicated. (**B**) Zoomed-in images of the areas around each mutation presented in ball and stick form to highlight the changes in residues. The mutated residues for the Q171R and Y65S mutants are highlighted in yellow while the mutated residue in C124S is encircled to exhibit how the oxygen molecule (red) was changed into a sulfur molecule (orange).

In order to analyze the contribution of each mutation on PTEN structure in greater detail, structural models of the PTEN novel mutants Q171R and Y65S were generated based on their DNA sequence using the wild-type PTEN (PDB ID: 5BUG) as a reference structure on the SWISS-MODEL web-based protein modeling platform. The structure of each resulting mutant model was then superimposed onto the wild-type PTEN structure, and the chain root-mean-square distance (RMSD)—the measure of the average distance between the backbone atoms of superimposed proteins, was computed using the BIOVIA Discovery Studio program (Figure 7). For better visualization of the possible structural changes brought about by the mutations, zoomed-in images of the ball and stick models of the mutations beside the wild-type forms are also presented. It is worth noting that the Q171R and Y65S mutations lie on structured domains of the PTEN protein and thus may appreciably affect the secondary structure of the protein despite the single amino acid substitution. RMSD values for mutant models overlaid onto the wild-type structure did not exceed the significance threshold of 0.15 as expected, since a single base change does not normally alter the overall protein structure significantly. The program used calculates for the average change throughout the entire protein so even with structural changes in the areas proximal to the mutated residues, the total calculated RMSD is expected to be low unless that particular residue is essential in providing a bond that holds the entire protein’s secondary structure together. The canonical mutant C124S had the highest RMSD, suggesting the highest overall structural deviation from the wild-type PTEN structure. While these results may indicate that these mutations do not affect overall protein structure, subtle structural changes at the active site and its surrounding residues may significantly affect catalytic activity which may cascade and be amplified in the context of signaling pathways. Thus, mutations in the proximity of the active center (D92, C124, R130; Y65S mutation) as well as the TI loop (residues 160–171; Q171R mutation) may have profound consequences on PTEN catalytic activity [42]. This may prove especially true for PTEN which acts as the only tumor suppressor in the PIK3CA pathway and is exemplified by the variety of PTEN mutations found in several cancers.

## 4. Discussion

PTEN is a well-characterized and important tumor suppressor with highly cited studies describing its role in tumor inhibition as far back as 1997 [43,44]. As the master regulator of the PI3K–Akt pathway, its role as an inhibitor of the pathway’s primary readouts of cell growth and survival has long been established. As a dual-phosphatase, PTEN enacts its function through both lipid-phosphatase activity, which is what it uses to dephosphorylate phosphatidylinositol-3,4,5 -trisphosphate and shut off PI3K–Akt signaling; and protein-phosphatase activity, which it uses to regulate several proteins such as FAK, SHC, PDGF-R, β-catenin, 5-HT2c-R or SMAD3, all of which play a role as mediators of cell migration, invasion and proliferation [1]. It was initially believed that PTEN mutations resulted in cancer development by way of dysregulation. More recent advances in sequencing technology, however, hint that oncogenesis as a result of PTEN mutations is not simply because of altered expression levels. Mutations within the coding region of PTEN may result in aberrant forms of the protein whose function is significantly hampered. These mutated PTEN proteins have even been demonstrated to interfere with the function of normal copies of the protein in a dominant negative fashion, sometimes resulting in an even more severe phenotype than if the PTEN genes in each cell were homogeneously mutated [13].

Single-nucleotide polymorphisms within the coding region of the PTEN gene may disrupt PTEN protein function and bring about oncogenesis in different ways. It has been demonstrated that different changes in the amino acids throughout the PTEN protein not only interfere with PTEN function but may also affect interactions with different downstream effectors and thus bring about different oncogenic readouts. For instance, the canonical mutant C124S harbors a mutation right in the center of the protein’s active site, thereby disrupting both the lipid-phosphatase and protein-phosphatase activity of the protein. The PTEN Y138L mutant, on the other hand, also has a mutation in its phosphatase domain but not exactly in its active site so only the lipid-phosphatase activity was compromised. Functionally, while both mutations were shown to be strong drivers of oncogenesis, they did so in vastly different ways as PTEN Y138L was still shown to be an inhibitor of the PI3K–Akt pathway while PTEN C124S lost that ability entirely [45]. Overall, this highlights the need to study each individual mutation independently, as the manner in which they drive oncogenesis may be different from each other and, in turn, would require a different therapeutic intervention.

In this study, cellular assays which measured key cancer hallmarks such as cellular proliferation, resistance to apoptosis, migratory capacity, and cytoskeletal remodeling were performed to assess the ability of the novel PTEN mutations Q171R and Y65S to confer an oncogenic cellular phenotype.

Prior to the actual functional assays, various bioinformatics methods were employed to provide some preliminary insight on the possible depth of impact of these mutations. The bioinformatics prediction tool PolyPhen-2 predicted both novel PTEN mutants Q171R and Y65S to be damaging to the function of the protein. The algorithm-based program Sorting Intolerant from Tolerant or SIFT also predicted that the novel and canonical mutants would have an effect on protein function. The novel Q171R mutant and canonical C124S mutant achieved SIFT scores of 0.04 while the novel mutant Y65S achieved a SIFT score of 0.00. A SIFT score is a normalized probability of observing the new amino acid at that position, and ranges from 0 to 1. A value of between 0 and 0.05 is considered to mean that the mutation would affect protein function. Finally, using the Align-GVGD bioinformatics program, all three mutations garnered high Grantham difference scores, which attempt to quantify the distance between two amino acids in an evolutionary sense and may possibly point to large-scale alterations in protein function. These predictions, while insufficient for definitively concluding the detrimental function of a given mutation, provided a valuable starting point by which to analyze the likely functional consequences of the novel mutations.

Based simply on the positions of the mutations in the PTEN protein itself, it can be inferred how overall function might be hampered. Both novel mutants Q171R and Y65S and the canonical mutant C124S bear substitutions in the phosphatase domain of the protein which exists between the amino acids 7-185 [46]. Both novel mutations present amino acid substitutions with changes to the properties of the amino acids present, with a shift from the neutral, polar glutamine to the basic, charged arginine in Q171R and a shift from the aromatic tryptophan to the polar serine in Y65S. Both could present significant changes to how these amino acids interact with the rest of the protein or possible ligands. The protein modeling, on the other hand, predicted low overall changes in the structures of the mutant proteins as compared to wild-type PTEN. None of the mutants garnered an RMSD value of 1.0 which would signify a significant deviation from the native protein structure [47]. This, by itself, is not surprising as all the mutations examined are single amino acid substitutions and should not affect the structure of the entire protein all too much. Interestingly, a deep mutation scanning study by Mighell et al. predicted Q171R as a damaging variant, being in the TI loop of the PTEN catalytic pocket. Y65S, on the other hand, was predicted to be neutral. Given that the humanized yeast assay used was very specific to the lipid phosphatase function of PTEN, it is likely that Y65S may affect another aspect of PTEN function [48].

The results of this study describe how the novel PTEN mutants Q171R and Y65S affect cellular proliferation, migration, resistance to apoptosis, and cytoskeletal organization. Similar to NIH3T3 cells transfected with the canonical PTEN mutant C124S, cells transfected with PTEN Q171R and Y65S displayed increased proliferation and migration rates. The CellTiter 96^®^ Aqueous One-Cell Proliferation Assay, which allows for counting the number of viable cells within a well, revealed that transfection of NIH3T3 cells with the mutant PTEN constructs significantly increased cellular proliferation after 72 h. Overexpression and knockdown studies conducted on Drosophila in the late 1990s displayed PTEN’s ability to inhibit cellular proliferation through both PI3K-dependent and PI3K-independent pathways [49,50]. This perspective has been reinforced by several studies conducted in both in vitro and in vivo mammalian systems, including some in the oncogenic context [51,52,53]. While proliferation is not known to be one of the major readouts of the PI3K–Akt pathway, its activation may also increase proliferation rates, especially in an oncogenic setting [54]. Furthermore, other studies have suggested that PTEN’s protein phosphatase activity may work in concert with its more characterized lipid phosphatase activity in regulating key cellular processes such as proliferation and migration. This may be due to its interactions with one or several of its proposed protein substrates, such as FAK, SHC, PDGF-R, β-catenin, 5-HT2c-R, or SMAD3, all of which play a role as mediators of cell migration, invasion, and proliferation [55].

The two novel mutants were also able to increase both migration rate as assessed in wound healing assays, and migratory potential as assessed by cytoskeletal reorganization and gross morphology changes. Similar to the canonical mutant C124S, both novel mutants significantly increased the rate of wound closure of NIH3T3 cells over a 20 h time frame. Several studies have described how loss of PTEN function can increase the migration of cells both in vitro and in vivo [56,57]. This may be due to the PI3K–Akt-independent function of PTEN. Interestingly, PTEN has been shown to interact with the metastasis suppressor protein 1 (*MTSS1*), the loss of which has been shown to result in a significant increase in cellular migration and invasion in pancreatic ductal adenocarcinoma cells [50]. The two proteins interact through PTEN’s protein phosphatase activity and this may potentially be the key to the correlation of PTEN loss with cell migration. Similarly, both PTEN Q171R and Y65S caused altered F-actin organization and morphological changes when transfected into NIH3T3 cells, compared to wild-type and vector-only controls. Morphological changes including cytoplasmic shrinkage, disorganized actin filaments, and the presence of cellular protrusions were apparent in all mutant setups [58]. Notable gross morphological changes such as cell rounding, refringency, and smaller cell size were also observed. These changes likely suggest an increased migratory potential as cytoskeletal reorganization and pseudopodial formation have been shown to be highly suggestive of a migratory phenotype, as these are among the preliminary steps taken by the cell to initiate movement [59].

In terms of the ability of each PTEN mutant to confer resistance to apoptosis, the results from the two assays conducted were only partially congruent. PTEN Q171R reduced apoptosis, as measured by both caspase-3/7 activity and Annexin V staining. PTEN Y65S, on the other hand, was found to increase caspase-3/7 activity but not apoptosis as measured by annexin V. Based on these findings, PTEN Q171R performed similarly to the canonical mutant PTEN C124S in conferring resistance to apoptosis. The results obtained on PTEN Y65S, on the other hand, are not necessarily conflicting as the Caspase-Glo^®^ 3/7 Assay simply measures caspase-3/7 activity and not apoptosis itself, unlike annexin V staining, which is known to be one of the gold standards of apoptosis assays. Caspases-3 and -7 are known as the executioner caspases and once fully activated, execute their protease activity on key cellular targets in order to trigger apoptosis. Even in their activated state, however, these proteins may be prevented from initiating apoptosis through the activity of a family of proteins known as IAPs or inhibitors of apoptosis that must be cleared by specific proteins such as Smac in order for apoptosis to proceed [60]. Interestingly, it has been shown that PTEN plays a role in Smac activation in a possible PI3K–Akt pathway-independent manner [61]. It may be possible that the mutation on PTEN Y65S allows it to activate the downstream cascade that leads to caspase-3/7 activation, but not the clearance of IAPs that would allow them to perform their function. Further studies on PTEN Y65S will clarify the extent of its effects on key cancer hallmarks.

This study took a reverse genetics approach by analyzing the phenotypic consequences of the novel mutations Q171R and Y65S on key cancer hallmarks. Western blot detection of Akt phosphorylation suggests that these novel mutants, similar to the canonical C124S mutant, modestly increase p-Akt levels. Whether these contribute to the phenotypes observed is as yet unknown. Further mechanistic studies need to build on these primary data, especially since PTEN also exerts many Akt-independent functions that may be relevant to cancer development and progression.

Going forward, functional and mechanistic studies may be carried out to (1) determine whether the Y65S and Q171R novel mutations interrupt one or both phosphatase functions of PTEN in *cis*; (2) whether they act in a dominant negative manner to inhibit PTEN wild-type function in *trans* via heterodimerization; (3) whether they engage different Akt-dependent and Akt-independent downstream pathways, with both overlapping and non-redundant oncogenic phenotypes; (4) whether they have different oncogenic potencies; and (5) whether the structural constraints imposed by the different mutations may have altered associations with downstream effectors. There is also a distinct possibility that the phenotypes observed may be the result of PTEN’s phosphatase-independent functions in the nucleus—something that cannot be totally ruled out at this point. Furthermore, the use of in vivo studies such as mouse models may help to determine whether these mutations have a role in colorectal cancer initiation and progression, as well as to further resolve their functional consequences in the context of an entire live organism.

This study has its own limitations, among which is the use of a heterologous model system in most of the assays. The choice of NIH3T3 over a normal colon cell line, however, was made not without careful consideration. These cells are a well-documented cell line used for studying and are predictive of phenotypic consequences of mutations in oncogenes and tumor suppressors. Unlike many human cell lines and primary cultures that require co-expression of cooperative oncogenes, NIH3T3 cells exhibit oncogenic phenotypes upon lone expression of a mutant oncogene or tumor suppressor [23,24]. Additionally, because PTEN mutations may not be typically associated with the initiating events in CRC, the use of normal colon cell lines may not be as physiologically relevant. The use of a colorectal cancer cell line, on the other hand, was precluded since oncogenic KRAS has repeatedly been shown to significantly downregulate the expression of PTEN [25]. This would have confounded the interpretation of observable phenotypes and would have made it difficult to decouple the effects of the PTEN mutants from those of PTEN downregulation as a result of an oncogenic Ras background. The results obtained should be verified in a homozygous wild-type KRAS-expressing colorectal cancer cell line such as SW48 HD PAR-006 from Horizon Discovery.

## Figures and Tables

**Table 1:** Primers used for generation of PTEN wild-type and mutant constructs. (*) Nucleotide in bold denotes the mutated nucleotide.

Primer	Primer Sequence *
PTEN WT	F: 5′-ATG ACA GCC ATC ATC AAA GAG ATC G-3′
	R: 5′-TCA GAC TTT TGT AAT TTG TGT ATG CTG ATC-3′
PTEN C124S (inner primers)	F: 5′-GCA GCA ATT CAC **A**GT AAA GCT GGA AAG G-3′
	R: 5′-CCC TTT CCA GCT TTA C**T**G TGA ATT GCT G-3′
PTEN Y65S (inner primers)	F: 5′-CAT AAA AAC CAT T**C**C AAG ATA TAC AAT C-3′
	R: 5′-GAT TGT ATA TCT TG**G** AAT GGT TTT TAT G-3′
PTEN Q171R (inner primers)	F: 5′-GGA GTA ACT ATT CCC AGT **G**AG AGG C-3′
	R: 5′-GCC TCT **C**AC TGG GAA TAG TTA CTC C-3′

**Table 2:** Predicted functional effects of PTEN mutations based on bioinformatics-based computations. The canonical PTEN C124S mutation and novel PTEN Q171R and Y65S mutations were all predicted to be damaging to PTEN function based on the bioinformatics prediction tools SIFT, PolyPhen-2, and Align GVGD.

Mutant	Classification	SIFT (Score)	PolyPhen-2 (Score)	Align GVGD (GV Score)
PTEN C124S	Canonical	Affect protein function (0.04)	Probably damaging (1.0)	Class C65 (111.67)
PTEN Q171R	Novel	Affect protein function (0.04)	Probably damaging (1.0)	Class C35 (42.81)
PTEN Y65S	Novel	Affect protein function (0.00)	Probably damaging (1.0)	Class C65 (143.11)

## Data Availability

All data generated or analyzed in this study are available from the corresponding author on reasonable request.

## Supplementary Materials

The following are available online at https://www.mdpi.com/article/10.3390/cells10123423/s1, Figure S1: Uncropped Western blot analysis.
