# Supplementary material for: The Novel Phosphatase Domain Mutations Q171R and Y65S Switch PTEN from Tumor Suppressor to Oncogene
Source: Cells. 2021 Dec 5;10(12):3423. doi: 10.3390/cells10123423 (PMC8700245; doi:10.3390/cells10123423)
Supplement: Supplementary file 1 [file cells-10-03423-s001.zip › cells-1487834-supplementary.pdf]

Supplementary File

The novel phosphatase domain mutations Q171R and Y65S switch PTEN from tumor suppressor to oncogene

Jose Antonio Ma. G. Garrido [1,§], Krizelle Mae M. Alcantara [1,‡,§], Joshua Miguel C. Danac [1], Fidel Emmanuel C. Serrano [1,†], Eva Maria Cutiongco-de la Paz [2,3] and Reynaldo L. Garcia [1,3,*]

[1] Disease Molecular Biology and Epigenetics Laboratory, National Institute of Molecular Biology and Biotechnology, University of the Philippines Diliman, Quezon City 1101, Philippines; jose_antonio_ma.garrido@upd.edu.ph (J.A.M.G.G.); KrizelleMae.Alcantara@nationwidechildrens.org (K.M.M.A.); jcdanac@up.edu.ph (J.M.C.D.); fidel.serrano@bzh.uni-heidelberg.de (F.E.C.S.)

[2] Institute of Human Genetics, National Institutes of Health, University of the Philippines Manila, Manila 1000, Philippines; eccutiongcodelapaz@up.edu.ph (E.M.C.-d.l.P.)

[3] Philippine Genome Center, University of the Philippines System, Diliman, Quezon City 1101, Philippines

[*] Correspondence: reygarcia@mbb.upd.edu.ph (R.L.G.); Tel.: +63 2 9818500 Loc. 3953

[‡] Present Address: Center for Gene Therapy, Abigail Wexner Research Institute, Nationwide Children's Hospital, 700 Children's Drive, Columbus, OH 43205, USA

[†] Present Address: Biochemistry Center, Hartmut Hoffmann-Berling International Graduate School of Molecular and Cellular Biology, University of Heidelberg, Im Neuenheimer Feld 328, 69120 Heidelberg, Germany

[§] These authors contributed equally to this work.

A

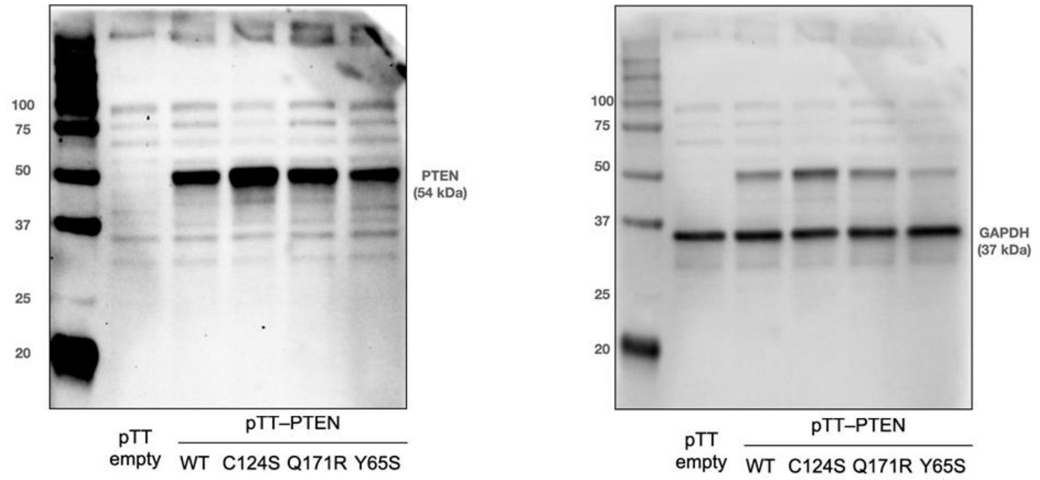

B

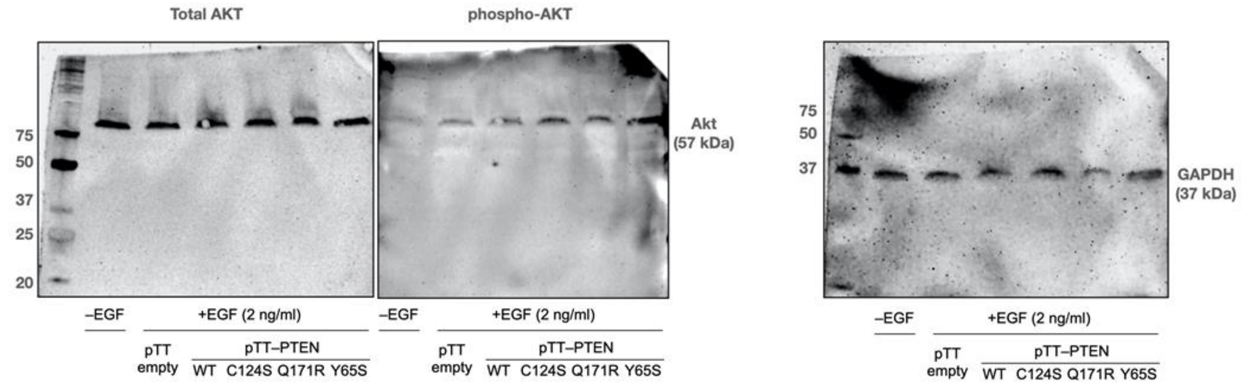

Figure S1. Uncropped western blot analysis showing: (A) exogenous expression of wild type and mutant PTEN constructs in NIH3T3 cells. The uncropped blot of PTEN (left) and GAPDH (right) are shown with the molecular weight markers. (B) Total Akt (left), phosphorylated Akt (middle) and GAPDH levels (right). pTT=empty pTargeT vector; wt=wild-type.
